# Efficacy of denosumab therapy for a 12-year-old female patient with Williams syndrome with osteoporosis and history of fractures: a case report

**DOI:** 10.1186/s13256-021-03175-9

**Published:** 2021-12-15

**Authors:** Masashi Uehara, Yukio Nakamura, Takako Suzuki, Noriko Sakai, Jun Takahashi

**Affiliations:** 1 Department of Orthopaedic Surgery, Shinshu University School of Medicine, 3-1-1 Asahi, Matsumoto, Nagano 390-8621 Japan; 2 Department of Human Nutrition, Faculty of Human Nutrition, Tokyo Kasei Gakuin University, 22 Sanban-cho, Chiyoda-ku, Tokyo, 102-8341 Japan; 3 Department of Orthopaedic Surgery, Nagano Children’s Hospital, 3100 Toyoshina, Azumino, Nagano 399-8288 Japan

**Keywords:** Bone mineral density, Denosumab, Fracture, Williams syndrome, Osteoporosis

## Abstract

**Background:**

A decrease in bone mineral density is common in patients with Williams syndrome. However, appropriate management for osteoporosis in Williams syndrome patients has not been established. We report the case of a 12-year-old female patient with Williams syndrome, who underwent denosumab treatment for osteoporosis.

**Case presentation:**

A 12-year-old Japanese female patient with Williams syndrome was shown to have very low bone mineral density. Bone mineral density was evaluated before treatment and at 5, 9, 17, 23, and 29 months of treatment by dual-energy X-ray absorptiometry. After denosumab therapy for 29 months, lumbar and total hip bone mineral density values had increased by 51.6% and 37.6%, respectively. No new fractures occurred during the observation period.

**Conclusions:**

To the best of our knowledge, this is the first experience with denosumab treatment in Williams syndrome patients with osteoporosis. Based on our findings, denosumab may be an effective treatment option for Williams syndrome patients with osteoporosis.

## Introduction

Williams syndrome is characterized by cardiovascular disease, distinctive facies and personality, mild intellectual disability, connective tissue abnormalities, growth retardation, and endocrine disorders [1–3]. Williams syndrome is a genetic multisystem disorder caused by chromosome 7 microdeletion [4]. The prevalence of this syndrome is estimated to be 1 in 10,000–15,000 people [5]. It is common for patients with Williams syndrome to have decreased bone mineral density (BMD), which means they are at higher risk for fractures [6–8]. However, the mechanism of BMD reduction in Williams syndrome has not yet been elucidated, and appropriate management therapy for osteoporosis in this syndrome has not been established.

There have been no reports of the use of bone resorption inhibitors, including denosumab, for Williams syndrome complicated by osteoporosis. We have demonstrated the safety and efficacy of denosumab in bisphosphonate-resistant cases in children and young adults [9–12]. Because it is an antibody preparation and, unlike bisphosphonate, does not accumulate, we used denosumab in this study. Since the use of denosumab in pediatric patients is off-label, consent was obtained from the patient and the patient's parents. We have also obtained approval from our hospital’s ethics committee for the use of denosumab in this case.

In this report, we describe the clinical results of a 12-year-old female patient with Williams syndrome accompanied by osteoporosis. Improvements in BMD and bone metabolic markers were observed over 2 years of denosumab treatment. Written consent for publication was obtained from the patient’s parents prior to the start of this study because she was too young and developmentally delayed.

## Case presentation

**Fig. 1:**
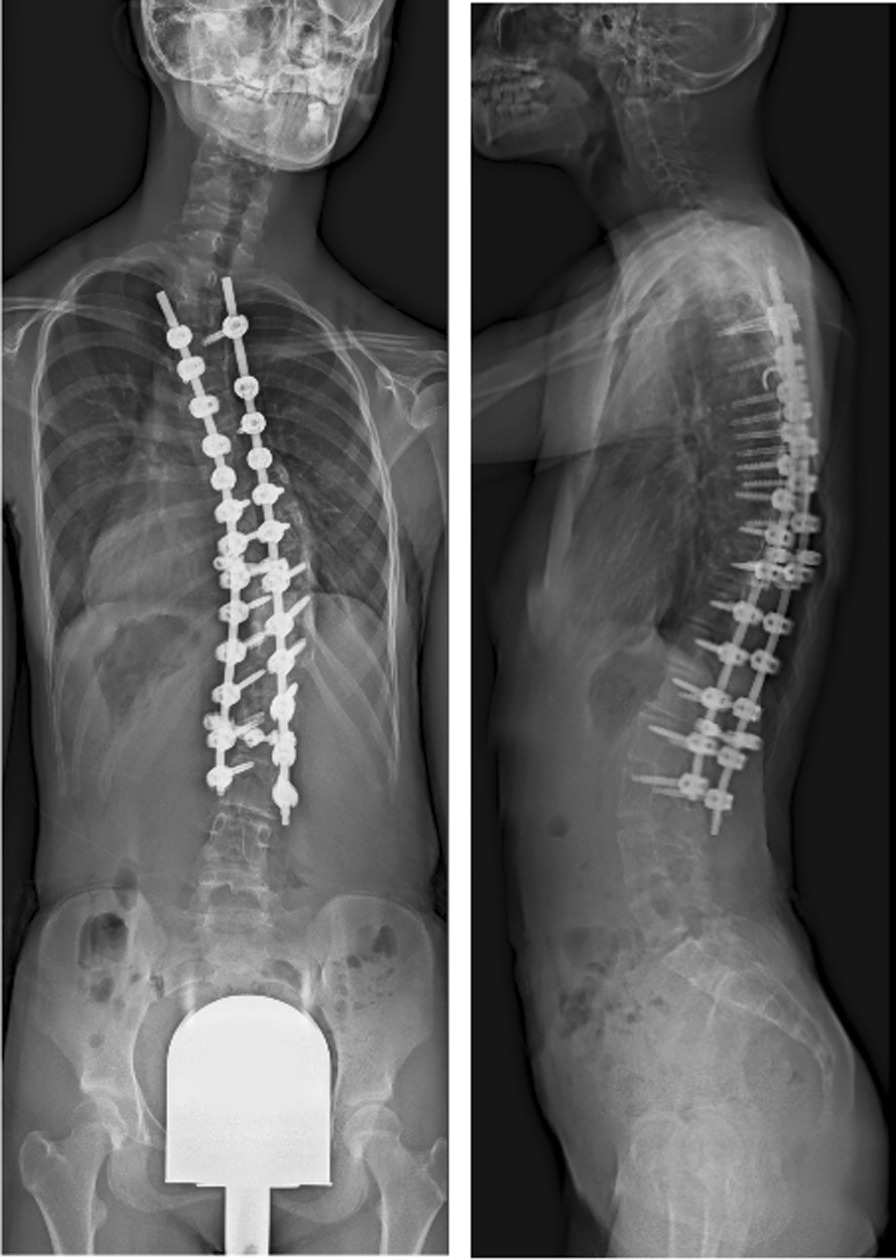
Radiographs of posterior spinal correction surgery at 10 months postoperatively. She underwent posterior spinal correction surgery for severe scoliosis at 12 years of age. Radiographs show **a** anterior–posterior view and **b** lateral view

An 11-year-old Japanese female patient with Williams syndrome was referred to our department for osteoporosis treatment. She has delayed development, characteristic face, and behavioral characteristics such as hypersensitivity to sounds in her infancy period. Collectively, she was diagnosed as Williams syndrome with a genetic deletion of 7q11. at 2 years old by a certified genetic expert at another institution. Her case was complicated by aortic stenosis that did not require surgery. She underwent correction surgery for scoliosis at 12 years old (Fig. 1). Her BMD and laboratory data are presented in Tables 1 and 2, respectively. Her reflexes in her extremities were mildly decreased, but her muscle strength was normal. She was able to walk steadily, and her gait was normal. The patient’s history revealed no previous fractures, and her family had no history of osteoporosis.

**Table 1.** Patient characteristic and bone mineral density in lumbar and total hip

	Age	Sex	BMI	Lumbar BMD (g/cm^2^)						Total hip BMD (g/cm^2^)					
				Before	5 months	9 months	17 months	23 months	29 months	Before	5 months	9 months	17 months	23 months	29 months
Case	12	F	15.0	0.562	0.695	0.745	0.763	0.860	0.852	0.540	0.599	0.668	0.655	0.744	0.743

*BMI* body mass index, *BMD* bone mineral density

**Table 2.** Patient laboratory data

	Before	1 month	2 months	5 months	9 months	11 months	17 months	23 months	29 months
Alb-corrected Ca	9.4	9.5	11.0	9.6	10.0	9.8	9.6	10.4	10.6
P	4.3	3.5	4.5	4.3	3.9	3.7	4.0	4.2	3.4
BAP	38.2	44.9	36.7	44.9	20.9	35.2	30.0	18.8	23.0
PINP	404	392	286	406	341	473	374	183	230
NTX	73.5	87.6	148	322	220	282	233	1596	1276
TRACP-5b	692	353	579	1290	652	1190	997	660	939
1,25(OH)2D	188	155	103	66.7	73.3	64.1	59.6	53.0	41.8
25(OH)D	13.7	N/A	N/A	20.8	22.8	25.4	24.3	20.5	20.0
Whole PTH	7.4	9.0	7.8	6.4	5.3	5.9	5.0	4.1	4.0

*Alb-corrected Ca* albumin-corrected calcium, *P* phosphorus, *BAP* bone-specific alkaline phosphatase, *PINP* type I procollagen N-terminal propeptide, *NTX* type I collagen amino-terminal telopeptide, *TRACP-5b* tartrate-resistant acid phosphatase 5b, *1,25(OH)2D* 1-α, 25-dihydroxyvitamin D3, *25(OH)D* 25-hydroxyvitamin D3, *PTH* parathyroid hormone

Osteoporotic treatment was initiated for the patient’s diminished lumbar and total hip BMD values at 12 years old. Subcutaneous injections of denosumab were given every 6 months since 2 months after surgery (12 years old), and the bone turnover markers and BMD values were examined before treatment and at 5, 9, 17, 23, and 29 months of treatment.

Serum tartrate-resistant acid phosphatase 5b (TRACP-5b) greatly decreased after the first administration (Table 2). Serum bone-specific alkaline phosphatase (BAP), type I procollagen N-terminal propeptide (PINP), whole parathyroid hormone (PTH), and 1-α, 25-dihydroxyvitamin D3 [1,25(OH)2D] decreased as well, while urinary type I collagen amino-terminal telopeptide (NTX) and 25-hydroxyvitamin D3 [25(OH)D] increased (Table 2). During the observation period, no calcium abnormalities such as hypocalcemia were observed (Table 2).

**Fig. 2:**
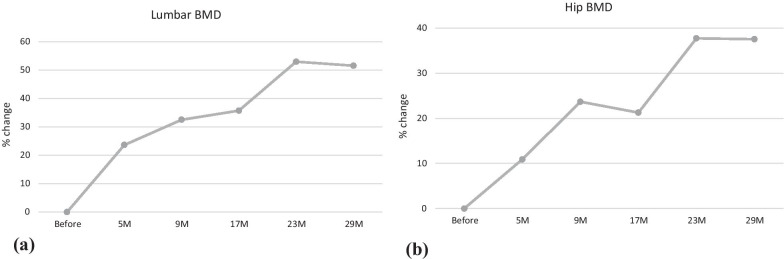
The change in bone mineral density (%). At 29 months of denosumab treatment, her lumbar and total hip bone mineral density increased by 51.6% and 37.6%, respectively

At 29 months of denosumab treatment, her BMD for lumbar and total hip increased by 51.6% and 37.6% at the end of our study, respectively (Fig. 2). There were no fractures or falls during the treatment period. She was able to keep active without getting out of shape through all the follow-up visits.

## Discussion

In this report, we evaluated the clinical course of patients treated with denosumab for Williams syndrome complicated by osteoporosis. Twenty-nine months after the start of treatment, the BMD values of the lumbar and total hip increased by 51.6% and 37.6%, respectively. No fractures or adverse effects such as hypocalcemia occurred during the therapy, and her bone metabolism markers were notably improved. To the best of our knowledge, this is the first study to describe the successful treatment of osteoporosis with denosumab in patients with Williams syndrome.

Decreased BMD is common in patients with Williams syndrome [6–8]. Stadi *et al.* reported that nearly 80% of Williams syndrome patients with a history of fractures showed an impaired bone mineral status [7]. However, they did not address treatment for osteoporosis. The causes of decreased BMD in Williams syndrome have not been elucidated yet, but may be multifactorial, including unknown bone characteristics in this syndrome and environmental factors such as lack of physical activity or poor nutrition [13–15]. Palmieri *et al.* showed that reduced renal tubular reabsorption of phosphate results in reduced BMD in adults with Williams syndrome [16]. In this case report, the patient was able to keep active without getting out of shape during the treatment period.

Denosumab is a monoclonal antibody against the anti-receptor activator of nuclear factor-kappa β ligand approved for the treatment of osteoporosis and the prevention of complications of skeletal metastases [17]. Huang *et al.* reported that denosumab is an available treatment for BMD reduction in pediatric cancer survivors [18]. We have also proven the efficacy and safety of denosumab in children and young adults with refractory osteoporosis [9–12], although there have been no reports on denosumab for Williams syndrome and osteoporosis. In our patient, BMD values were very low. As a result, we started denosumab treatment to prevent ensuing fractures and improve BMD after a careful discussion with her and her family. At 29 months of denosumab treatment, both lumbar and total hip BMD values increased, and there were no additional fractures.

Markers of bone resorption are frequently used to assess the effectiveness of osteoporosis treatment [19]. BAP decreased to approximately 21% with denosumab, while PINP decreased to approximately 7% with increase in NTX and TRACP-5b. These results suggested that denosumab slightly inhibited bone resorption and increased bone mineral density in patients with Williams syndrome with osteoporosis.

Hypercalcemia occurs in 15–50% of children with Williams syndrome [20, 21]. In our case, calcium abnormality was not observed during the observational period. Vitamin D sensitivity [22] and decreased 1,25(OH)2D [23] were proposed mechanism of hypercalcemia in Williams syndrome. In this case, 1,25(OH)2D was decreased, but hypercalcemia did not occur.

The limitations of this study are that the long-term effects of denosumab on Williams syndrome with osteoporosis have not been confirmed and the sample size is small. Furthermore, further evaluation of this case is needed to validate our results. Because bone metabolism in Williams syndrome itself is not well known, it is difficult to compare our findings with those of previous pediatric patients. Nevertheless, bone density has increased, there have been no fractures during treatment, and patient satisfaction has been high with no adverse events.

## Conclusion

After 29 months of treatment with denosumab, BMD levels and bone turnover markers improved in patients with Williams syndrome who had osteoporosis. The drug therefore represents an effective treatment option in such cases and warrants further study.

## Data Availability

The clinical and imaging data supporting the analysis and findings of this study will be available from the corresponding author upon reasonable request.

## Abbreviations

BMD: Bone mineral density
TRACP-5b: Tartrate-resistant acid phosphatase 5b
BAP: Bone-specific alkaline phosphatase
PINP: Type I procollagen N-terminal propeptide
PTH: Parathyroid hormone
1,25(OH)2D: 1-α, 25-dihydroxyvitamin D3
NTX: Type I collagen amino terminal telopeptide
25(OH)D: 5-Hydroxyvitamin D3
